# Added Value of Shear Wave Elastography for Noninvasive Detection of Axillary Lymph Node Metastasis and IHC-Defined Surrogate Subtype Characterization in Breast Cancer

**DOI:** 10.3390/cancers18213549

**Published:** 2026-11-04

**Authors:** Jie Chen, David P. Rosen, Tanin Adl Parvar, Robert T. Fazzio, Nicholas B. Larson, Mostafa Fatemi, Azra Alizad

**Affiliations:** 1 Department of Radiology, Mayo Clinic College of Medicine, 200 1st Street SW, Rochester, MN 55902, USA; cjie928@outlook.com (J.C.); adlparvar.tanin@mayo.edu (T.A.P.); fazzio.robert@mayo.edu (R.T.F.); 2 Department of Physiology and Biomedical Engineering, Mayo Clinic College of Medicine, Rochester, MN 55902, USA; rosen.david@mayo.edu (D.P.R.); fatemi.mostafa@mayo.edu (M.F.); 3 Department of Quantitative Health Science, Mayo Clinic College of Medicine, Rochester, MN 55902, USA; larson.nicholas@mayo.edu

**Keywords:** axillary lymph node, breast cancer, lymph node metastasis, machine learning, nodal staging, shear wave elastography, ultrasound

## Abstract

**Background:** Accurate preoperative characterization of axillary lymph nodes (ALNs) is important in breast cancer, but conventional ultrasound has variable diagnostic performance. We evaluated quantitative shear wave elastography (SWE) for distinguishing reactive from metastatic ALNs, and we explored associations between nodal SWE parameters, breast cancer subtypes, and histological grades. **Methods:** We prospectively enrolled women with suspicious breast lesions and ALNs identified by clinical ultrasound who were scheduled for biopsy. After exclusions, 141 patients (57 reactive and 84 metastatic ALNs) were included. ElasticNet-regularized logistic regression with nested cross-validation compared clinical ultrasound alone with ultrasound plus SWE. Among 70 patients with metastatic ALNs and immunohistochemical characterization, SWE parameters were compared across surrogate subtypes and histological grades using Kruskal–Wallis tests with Holm adjustments. **Results:** The combined ultrasound–SWE model achieved an area under the receiver operating characteristic curve of 0.911 (95% CI, 0.859–0.961) compared to 0.798 (95% CI, 0.723–0.873) for ultrasound alone. At the Youden-derived threshold, the combined model achieved 88.1% sensitivity, 82.5% specificity, and 85.8% accuracy. Among metastatic ALNs, SWE parameters, namely V_mean_, V_sd_, and V_max_, differed significantly across surrogate subtypes, with the most pronounced differences observed between Luminal A and Luminal B tumors, (*p* = 0.0250, *p* = 0.0069, and *p* = 0.0065). No statistically significant differences were detected with histological grades in the five SWE parameters. **Conclusions:** Quantitative SWE complemented conventional ultrasound for assessing axillary nodal metastasis, with higher cross-validated discrimination for the combined model. Exploratory analyses identified differences in several SWE stiffness metrics between Luminal A and Luminal B tumors; these subtype-associated biomechanical differences require validation in larger cohorts.

## Simple Summary

Assessing whether breast cancer has spread to the axillary lymph nodes is important for determining the disease stage and guiding treatment. Standard ultrasound is commonly used to evaluate lymph nodes, but it cannot always reliably distinguish cancerous lymph nodes from those enlarged due to benign reactive changes. In this study, we evaluated shear wave elastography, an ultrasound-based technique that measures tissue stiffness, in 141 women with breast cancer undergoing lymph node biopsy. Adding lymph node stiffness measurements to standard ultrasound improved the ability to distinguish cancerous from non-cancerous lymph nodes. Lymph node stiffness also differed among some breast cancer subtypes, particularly Luminal A and Luminal B tumors, but it was not associated with tumor grade. These findings suggest that shear wave elastography may provide useful additional noninvasive information for evaluating axillary lymph nodes in patients with breast cancer.

## 1. Introduction

Breast cancer is the second leading cause of cancer incidence in the United States, and it was the most commonly diagnosed cancer among women in 2024 [1]. In breast cancer, assessing axillary lymph node (ALN) status is crucial for accurate staging, prognosis, and treatment planning, as ALNs are often the primary route of regional metastasis from the primary tumor [2]. Clinical assessments of ALNs often lack sufficient accuracy for the diagnosis of metastatic ALNs and are associated with very low sensitivity (30%) [3]. Pathological examinations of core needle biopsy (CNB) and surgical pathology are established axillary evaluation methods and remain the gold standard for the definitive diagnosis of ALNs [4,5]. Axillary lymph node dissection and sentinel lymph node biopsy are commonly used methods for diagnosis; however, despite their high accuracy and diagnostic value, they are invasive procedures that may lead to complications, such as infection, nerve damage, and arm lymphedema [6,7]. Furthermore, lymph node metastasis is an important determinant of tumor staging and treatment planning, and it may influence the indication for systemic chemotherapy [8]. Noninvasive imaging approaches have therefore been investigated to detect nodal spread while reducing the reliance on invasive assessments [9]. Developing reliable, noninvasive methods for detecting lymph node metastasis is clinically important for improving axillary lymph node assessment, increasing diagnostic accuracy, and guiding precise, individualized treatment strategies [10].

Ultrasound (US) is routinely used to assess axillary lymph nodes (ALNs) in breast cancer, as it is a noninvasive, radiation-free imaging modality. However, its diagnostic performance remains variable, with reported sensitivity and specificity of 26.4–92.0% and 55.6–98.1%, respectively [11,12]. Other techniques, such as Magnetic Resonance Imaging, Computed Tomography, and Positron Emission Tomography, provide complementary metabolic or cross-sectional information, but they have limited utility for the primary assessment of ALNs [13]. Given these limitations, additional techniques are needed to improve ALN classification. It is known that most malignant lesions exhibit higher stiffness than those that are benign [14]. Shear wave elastography (SWE), a tool for measuring tissue stiffness, has shown efficacy for the differentiation of malignant from benign lesions, the prediction of ALN metastasis and tumor aggressiveness, and the assessment of responses to neoadjuvant therapy in breast cancer patients [15,16,17,18,19,20].

Previous studies have shown that SWE can improve the differentiation of benign and metastatic ALNs when combined with conventional ultrasound [21,22,23]. However, the incremental value of quantitative SWE beyond routinely assessed ultrasound features and its relationship with breast cancer biology remain incompletely characterized. Tissue stiffness reflects extracellular matrix remodeling and has been associated with breast cancer aggressiveness [24,25]; prior SWE studies have also reported associations between stiffness and IHC-defined surrogate subtypes [18,26,27]. In addition to histological grade and ALN involvement, immunohistochemical (IHC) profiling provides important biological context for ALN assessment. IHC markers, including estrogen receptors (ERs), progesterone receptors (PRs), human epidermal growth factor receptor 2 (HER2), and Ki-67, are routinely evaluated to classify breast cancers into IHC-defined surrogate subtypes, with distinct prognostic and therapeutic implications [19,26,27,28].

We therefore hypothesize that metastatic ALNs from different breast cancer subtypes may exhibit distinct biomechanical properties. The primary objective of this study was to evaluate the added value of quantitative SWE beyond conventional ultrasound for distinguishing reactive from metastatic ALNs. As a secondary exploratory objective, we examined whether SWE parameters in metastatic ALNs differed by surrogate breast cancer subtypes and histological grades.

## 2. Materials and Methods

### 2.1. Ethical Statement

This prospective study was approved by the institutional review board (IRB #13-006035) and was compliant with the Health Insurance Portability and Accountability Act. Written informed consent was obtained from all participants before conducting the research study.

### 2.2. Participants

Patient volunteers meeting the eligibility criteria were prospectively and consecutively enrolled between 2014 and 2024 after identification of an ALN through clinical ultrasound and a referral for image-guided biopsy. The ultrasound equipment, SWE software, and image acquisition protocol remained consistent throughout the study period. The study included sonographically identified ALNs referred for biopsy, providing a histopathologic ground truth. Patients with benign breast pathology (n = 24), lymphoma (n = 1), or chronic lymphocytic leukemia (n = 1) were excluded. The remaining 141 patients with primary breast cancer were included in the ALN classification analysis. Twenty-seven of these cases had incomplete IHC data, leaving 114 cases for descriptive analyses of primary-tumor IHC and histopathologic characteristics; among these, 70 had metastatic ALNs and were included in analyses relating to nodal SWE measurements of molecular subtypes and histological grades (Figure 1).

### 2.3. Ultrasonographic and SWE Assessments

All enrolled patients underwent clinical breast and axillary lymph node US as part of routine clinical care. Morphologic ALN features of cortical thickness, fatty hilum status, shape, and boundary definition were considered. B-mode US and SWE were performed using a GE LOGIQ E9 system with a 2–8 MHz linear-array transducer (9L-D; GE Medical Systems, Milwaukee, WI, USA). Two experienced breast imaging sonographers (15–25 years of US experience and more than 6 years of SWE experience) performed the examinations. The target ALN was centered on the image. To reduce motion artifacts, participants suspended respiration for approximately 3 s during each acquisition, and minimal transducer compression was used to limit precompression effects [29].

For each participant, B-mode imaging was followed by six SWE acquisitions obtained in the plane showing the greatest cortical enlargement. Shear wave speed was measured with the scanner’s 2D dual-circle tool, allowing for simultaneous visualization of the region of interest (ROI) on the B-mode image and SWE map. Up to three non-overlapping circular ROIs (3 mm in diameter) were placed within the enlarged nodal cortex. Measurements from at least three SWE maps were averaged to calculate SWE parameters of the ALN. V_mean_, V_max_, and V_min_ represent the mean, maximum, and minimum all measured velocity values, respectively, whereas V_sd_ is the standard deviation of the elastography pixel values within a given ROI and reflects the spatial heterogeneity of velocity. The f_mass_ parameter was included because prior work has suggested that it may provide additional value for characterizing primary breast tumors in relation to immunohistochemical subtypes and ALN status [19]. As described by Gu et al. [19], f_mass_ was calculated as f_mass_ = 1000 V_min_/d, where V_min_ is the minimum shear wave speed (m/s) and d is the maximum lesion dimension (mm). The physical rationale for f_mass_ is that the shear wave speed may be underestimated in small lesions when lesion dimensions are comparable to the shear wavelength. By weighting the minimum shear wave speed by the inverse of the lesion diameter, f_mass_ is intended to partially account for this size-dependent effect. Additional details regarding the derivation and rationale for f_mass_ are provided by Gu et al. [19]. For ALNs, d corresponded to the long-axis diameter, defined as the largest nodal dimension on B-mode imaging. From the clinical ultrasound imaging, the fatty hilum, cortical thickness, shape, and boundaries were recorded for analysis.

### 2.4. Ultrasound-Guided Lymph Node Biopsy

Image-guided biopsy of the target ALN was performed by a board-certified radiologist after SWE acquisition, typically on the same day. Only ALNs with a definitive pathology result, classified as positive or negative for malignancy, were included in the analysis. The pathology result of the sampled ALN served as the reference standard for metastatic status.

### 2.5. Histopathological Data and Immunohistochemical Markers

ALN metastatic status was assigned from the pathology result of the biopsy-sampled node. Histological type, histological grade, and IHC biomarker results for the primary breast cancer were obtained from the clinical record and breast biopsy pathology. ERs and PRs were considered positive when at least 1% of invasive tumor cells showed nuclear immunoreactivity. HER2 was classified according to ASCO/CAP criteria [30]: IHC scores of 0 or 1+ were negative, 3+ was positive, and 2+ results were adjudicated by fluorescence in situ hybridization (FISH). Ki-67 was recorded as the percentage of positively stained tumor nuclei. For consistency with the prespecified St Gallen surrogate classification used in this study [31], tumors were categorized into five IHC-defined groups: Luminal A and ER-positive/HER2-negative, with Ki-67 <14%; Luminal B/HER2-negative and ER-positive/HER2-negative, with Ki-67 ≥ 14%; Luminal B/HER2-positive and ER-positive/HER2-positive, regardless of Ki-67; HER2-positive, non-Luminal, and ER-negative/PR-negative/HER2-positive; and triple negative as ER-negative/PR-negative/HER2-negative. These categories represent IHC surrogate subtypes rather than intrinsic molecular subtypes determined by gene expression profiling.

### 2.6. Statistical Analysis Methods

Statistical analyses were performed using R version 4.1.3 (R Core Team, Vienna, Austria). Continuous variables were summarized as mean ± standard deviation and compared between reactive and metastatic ALNs using the Mann–Whitney U test [32]. Categorical variables were summarized as counts and percentages and compared using the chi-square test. For the set of univariable group comparisons in Table 1, *p*-values were adjusted using the Holm procedure [33].

Diagnostic modeling of lymph node metastasis using SWE and clinical ultrasound features was performed using logistic regression. Given the sample size and number of candidate predictors, measures were taken to reduce the risk of model overfitting. Elastic-Net regularization [34] was applied to the logistic regression model to perform regularized feature selection and reduce model complexity. Model development and internal validation were performed using nested cross-validation [35], thereby separating hyperparameter tuning from performance estimation. Specifically, 10-fold cross-validation was used in the inner loop for hyperparameter tuning, while leave-one-out cross-validation (LOOCV) was used in the outer loop to estimate model performance on held-out observations. Model discrimination was summarized using the area under the receiver operating characteristic curve (AUC), with 95% confidence intervals estimated using an influence-curve-based method [36]. Sensitivity, specificity, positive predictive value, negative predictive value, and accuracy were calculated from the LOOCV predictions. The optimal classification threshold was determined from the pooled LOOCV predictions using the Youden criterion [37].

To assess the incremental discriminative value of SWE beyond clinical ultrasound, two models were evaluated using the same nested cross-validation framework: (1) clinical ultrasound parameters alone and (2) clinical ultrasound plus SWE parameters. The difference between the paired pooled AUCs was assessed using a nonparametric permutation test based on the out-of-sample predictions. For each permutation, the two model predictions were randomly exchanged within each observation, preserving the paired structure and observed outcome labels, and the difference between the resulting pooled AUCs was recalculated. The permutation *p*-values were obtained by comparing the observed AUC difference with the null distribution of AUC differences generated by these within-observation permutations [38]. All 141 observations had complete values for the predictors used in the modeling dataset; no predictor–value imputation was required.

Associations between SWE parameters in metastatic lymph nodes and histopathological characteristics of the primary tumor, including immunohistochemical (IHC)-Defined surrogate subtypes and histological grades, were evaluated exclusively among the 70 patients with metastatic ALNs using nonparametric methods. For each of the five SWE parameters, a Kruskal–Wallis test [39] was first performed as an omnibus test, and a Holm correction was applied to the calculated *p*-values for each SWE parameter. Subsequent post hoc pairwise comparisons were conducted using the Conover–Iman test [40]. Because the Conover–Iman test corrects only for multiple comparisons within a single SWE parameter, an additional Bonferroni correction for was applied to the *p*-values produced by the Conover–Iman test. A Bonferroni correction was applied instead of an additional Holm correction because the Holm correction relies on the ordering of *p*-values, and the potential statistical effects of nesting multiple Holm corrections in this scenario are opaque. The Bonferroni correction was calculated as ***p_adj_*** = **min**(1, ***p*** ∗ ***M***), where ***M*** is the number of SWE parameters. In this case ***M*** = 5, as we considered five SWE parameters (V_mean_, V_sd_, V_max_, V_min_ and f_mass_).

## 3. Results

### 3.1. ALN Classification Results

Of the 141 patients included in the study, the histopathology results of the ALNs indicated that 57 ALNs were negative for metastasis (reactive) and 84 were positive for metastatic carcinoma. Patient ages ranged from 28 to 89 years. The mean age was 58.24 ± 12.81 years in the metastatic group and 58.25 ± 12.00 in the reactive group. The presence or absence of fatty hilum, cortical thickness, shape, and boundary differed significantly between metastatic and reactive ALNs (all *p*-values < 0.05; Table 1).

### 3.2. Machine Learning Model for Detection of Metastatic Lymph Node

We evaluated the classification performance of the models using ROC analysis. The highest performing model was the combined SWE and clinical ultrasound model, which achieved an AUC of 0.911 (CI 0.859–0.961), outperforming clinical ultrasound alone with an AUC of 0.798 (CI 0.723–0.873). The combined SWE and clinical ultrasound model demonstrated a sensitivity of 0.881, specificity of 0.825, PPV of 0.881, NPV of 0.825, and overall accuracy of 0.858, all of which were numerically higher than those of clinical ultrasound-only model (Table 2). Table 2 summarizes the nested cross-validation ROC performance metrics for conventional ultrasonography and SWE in differentiating between reactive and metastatic ALNs using machine learning models. The corresponding ROC curves based on pooled LOOCV results are presented in Figure 2. The results of the non-parametric permutation test, presented in Table 2, demonstrate a statistically significant difference between the AUCs of the model using clinical ultrasound features alone and the model combining clinical ultrasound with SWE parameters.

### 3.3. Relationships Between SWE Parameters of ALN and IHC Markers of Breast Cancer

Table 3 shows the counts and percentages of cancer grades and IHC markers, as well as the histological types of breast cancer, in the group with metastatic ALNs of this study.

Table 4 summarizes the associations between SWE metrics in metastatic lymph nodes and the expression of IHC biomarkers, IHC-defined surrogate subtypes, and histological grades, and it reports the *p*-values for the corresponding Kruskal–Wallis tests. The *p*-values of the Kruskal–Wallis test for the five SWE parameters detected statistically significant differences between IHC-defined surrogate subtypes at a *p*-value threshold of *p* < 0.05 for V_mean_, V_sd_, and V_max_. No statistically significant differences were detected for histological grades in the five SWE parameters.

The *p*-values for subsequent pairwise comparisons between molecular subtypes in V_mean_, V_sd_, and V_max_ are presented in Table 5. The lower stiffness metrics observed in metastatic lymph nodes with Luminal A compared to Luminal B subtypes in these three metrics (Table 4) were found to be statistically significant (Table 5). Specifically, V_mean_, V_sd_, and V_max_ were significantly different between Luminal A and HER2-positive Luminal B (*p* = 0.0018, *p* = 0.0041, and *p* = 0.0009, respectively) and between Luminal A and HER2-negative Luminal B (*p* = 0.0061, *p* = 0.0002, and *p* = 0.0002, respectively). Additionally, the lower V_sd_ observed Luminal A compared to HER2-positive tumors (Table 4) was also determined to be statistically significant (*p* = 0.046, Table 5), whereas differences in V_mean_ and V_max_ for this comparison did not reach statistical significance.

No statistically significant differences were observed between the non-Luminal A subtypes. Comparisons between HER2-positive Luminal B and HER2-negative Luminal B, as well as between Luminal B subtypes and HER2-positive or triple-negative (TN) disease, showed no significant differences across V_mean_, V_sd_, or V_max_ (Table 5, all *p* ≥ 0.46). Similarly, comparisons involving HER2-positive and TN subtypes did not demonstrate significant differences for any SWE parameter.

Overall, these findings indicate that SWE stiffness metrics in metastatic lymph nodes differ primarily between Luminal A and Luminal B molecular subtypes, while SWE parameters appear largely comparable among Luminal B, HER2-positive, and TN subtypes.

### 3.4. Representative Images of SWE Images of Reactive and Metastatic ALNs

Representative B-mode US and SWE images of reactive and metastatic ALNs are presented in Figure 3 and Figure 4. Figure 3 represents two larger ALNs. At the top, images (A and B) present a reactive ALN from a 45-year-old woman with invasive mammary carcinoma, mixed ductal and lobular features, grade (G) III, and an IHC-defined surrogate subtype classification of triple-negative (TN) breast cancer. The lymph node exhibited slight cortical thickening with a preserved and prominent hilum on clinical ultrasound, while the SWE map demonstrated low cortical stiffness. Histopathological results showed no evidence of malignancy, confirming the ALN as reactive. Figure 3C,D represent a metastatic ALN from a 56-year-old woman with invasive ductal carcinoma (grade II), with the IHC-defined surrogate subtype classified as Luminal B and HER2-negative. The lymph node demonstrated marked cortical thickening with an almost absent hilum, whereas the SWE map revealed high cortical stiffness, and the biopsy confirmed an ALN originating from breast cancer.

Figure 4 represents two smaller ALNs. At the top of the figure, images (A and B) present a reactive ALN from a 56-year-old woman with IDC GII, with the IHC-defined surrogate subtype classified as TN. The ALN exhibited a slight cortical thickness on the clinical ultrasound while the SWE map demonstrated low cortical stiffness. Biopsy showed no evidence of malignancy, confirming the ALN as reactive. On the bottom of Figure 4, images (C and D) are from a 76-year-old woman with invasive ductal carcinoma (grade II), with the IHC-defined surrogate subtype classified as Luminal B with HER2 Neg. On B-mode US, the lymph node demonstrated marked cortical thickening with a nearly absent hilum, whereas the SWE map revealed high cortical stiffness. A biopsy confirmed a metastatic ALN originating from primary breast cancer.

## 4. Discussion

In this study, we investigated the diagnostic performance of SWE as an adjunct to conventional ultrasound for the characterization of axillary lymph nodes (ALNs) in a cohort of patients with breast cancer. The principal finding was the substantial improvement in cross-validated discrimination when SWE parameters were added to conventional ultrasound features, with the AUC increasing from 0.798 for conventional ultrasound alone to 0.911 for the combined model. Consistent with this improvement in discrimination, the combination of SWE and conventional ultrasound demonstrated higher sensitivity, specificity, and overall diagnostic accuracy than conventional ultrasound alone. These findings suggest that SWE provides incremental diagnostic information beyond that available from conventional sonographic assessment.

The improved performance of the combined approach may reflect the complementary information provided by the two techniques. Conventional ultrasound primarily characterizes morphologic features associated with nodal involvement, including cortical thickness, fatty hilum, shape, and nodal margins. In our cohort, the presence or absence of the fatty hilum, cortical thickness, shape, and boundary differed significantly between metastatic and reactive ALNs, consistent with previous studies [41,42]. However, morphologic abnormalities may overlap between reactive and metastatic nodes, potentially limiting the specificity of conventional ultrasound. SWE provides additional quantitative information regarding tissue mechanical properties, including stiffness and stiffness heterogeneity, which may reflect structural changes associated with metastatic infiltration. Thus, integrating morphologic and biomechanical information may provide a more comprehensive characterization of suspicious ALNs than either approach alone. Our findings are consistent with previous reports [43,44] that demonstrate improved diagnostic performance when SWE is combined with conventional ultrasound compared to either modality alone.

From a clinical perspective, these results support SWE as a potentially useful adjunct to conventional ultrasound rather than a replacement for established axillary imaging or tissue-sampling approaches. In patients with sonographically identified ALNs, quantitative SWE measurements may provide additional information for estimating the likelihood of metastatic involvement and could potentially contribute to decisions regarding which nodes warrant image-guided sampling. However, the improvement in cross-validated AUCs observed in the present study should not be interpreted as evidence that the combined model is ready for clinical implementation. Independent validation in larger, multi-center populations is required to determine whether this diagnostic improvement is reproducible across different patient populations, ultrasound systems, operators, and institutional practices and whether it provides meaningful benefit within existing clinical axillary evaluation pathways.

The characteristics of our study population are also important when considering the potential clinical application of these findings. The cohort was enriched for patients with sonographically identified ALNs referred for image-guided biopsy and therefore does not represent an unselected breast cancer population undergoing routine axillary staging. This selection was necessary because biopsy histopathology served as the reference standard for validating SWE findings; histopathologic confirmation would generally not have been available for patients without a clinical indication for biopsy. Consequently, the prevalence of nodal metastasis in our cohort was relatively high, which may particularly influence predictive values and limits extrapolation to populations with a lower prevalence of nodal disease. Our findings are therefore most directly applicable to the characterization of sonographically identified ALNs being considered for biopsy rather than to screening or routine axillary staging of an unselected breast cancer population.

As a secondary exploratory analysis, we also examined whether SWE characteristics of metastatic ALNs differ according to IHC-defined surrogate breast cancer subtype. Metastatic ALNs associated with Luminal A tumors demonstrated lower stiffness measurements than those associated with Luminal B/HER2-positive tumors across several SWE metrics. A modest difference in stiffness heterogeneity (V_sd_) was also observed between Luminal A and HER2-positive tumors, whereas differences in V_mean_ and V_max_ were not significant, and no consistent differences were identified among the non-Luminal A subtypes. Previous studies have associated SWE stiffness of primary breast tumors with histopathologic characteristics, IHC-defined subtype, and axillary lymph node status [18,26,45,46]. The less consistent subtype-related differences observed in metastatic ALNs may reflect differences between the primary-tumor and nodal microenvironments. Metastatic breast cancer cells can alter the lymph node extracellular matrix, including increasing nodal collagen density [47], which may contribute to changes in nodal mechanical properties. However, given the small numbers within individual molecular subtype groups, these analyses should be considered hypothesis-generating. The present findings do not establish SWE as a method for differentiating IHC-defined surrogate subtypes of metastatic ALNs; rather, they suggest that potential subtype-related differences in nodal biomechanical properties warrant further investigation in larger, adequately powered cohorts. Accordingly, the subtype findings should be interpreted as secondary to the primary diagnostic finding of improved discrimination between metastatic and reactive ALNs when SWE is added to conventional ultrasound.

Several limitations should be considered when interpreting our findings. First, this was a single-center study with a limited overall sample size, which may restrict the generalizability and precision of the diagnostic performance estimates. The small numbers within individual IHC-defined surrogate subtype groups further limited statistical power for the exploratory subtype analyses and increased the possibility that observed differences may be unstable. Also, the study included sonographically identified ALNs referred for biopsy to provide histopathologic ground truth. The higher prevalence of nodal metastasis should be considered when applying predictive values to lower-prevalence populations. Second, although cross-validation was used to estimate model performance and reduce optimism associated with evaluation in the development dataset, the combined ultrasound–SWE approach was not tested in an independent external cohort. Therefore, the observed increase in AUCs from 0.798 to 0.911 requires independent validation before conclusions regarding clinical utility can be drawn. Third, SWE measurements may be influenced by ultrasound platform characteristics, acquisition technique, operator experience, lymph node depth and size, and other technical factors. Reproducibility across different equipment, operators, and institutions therefore remains to be established. Finally, the relatively long recruitment period may have introduced temporal variability related to imaging equipment, acquisition practices, patient selection, or clinical management.

## 5. Conclusions

In conclusion, quantitative SWE complemented conventional ultrasound for noninvasive assessment of axillary nodal metastasis in this prospective single-center breast cancer cohort, with higher cross-validated discrimination from the combined model than from clinical ultrasound alone. These findings suggest that quantitative assessment of tissue stiffness and heterogeneity may provide complementary information to conventional sonographic morphology and support further investigation of SWE as an adjunctive tool for evaluating sonographically identified ALNs in patients with breast cancer. The observed associations between SWE parameters and IHC-defined surrogate subtypes are exploratory and require confirmation in larger cohorts. Prospective, independent multi-center studies are needed to validate the incremental diagnostic performance of SWE, establish reproducibility across imaging platforms and operators, and determine whether incorporating SWE into conventional ultrasound assessment can meaningfully improve clinical axillary evaluation and biopsy decision-making.

## Figures and Tables

**Figure 1:**
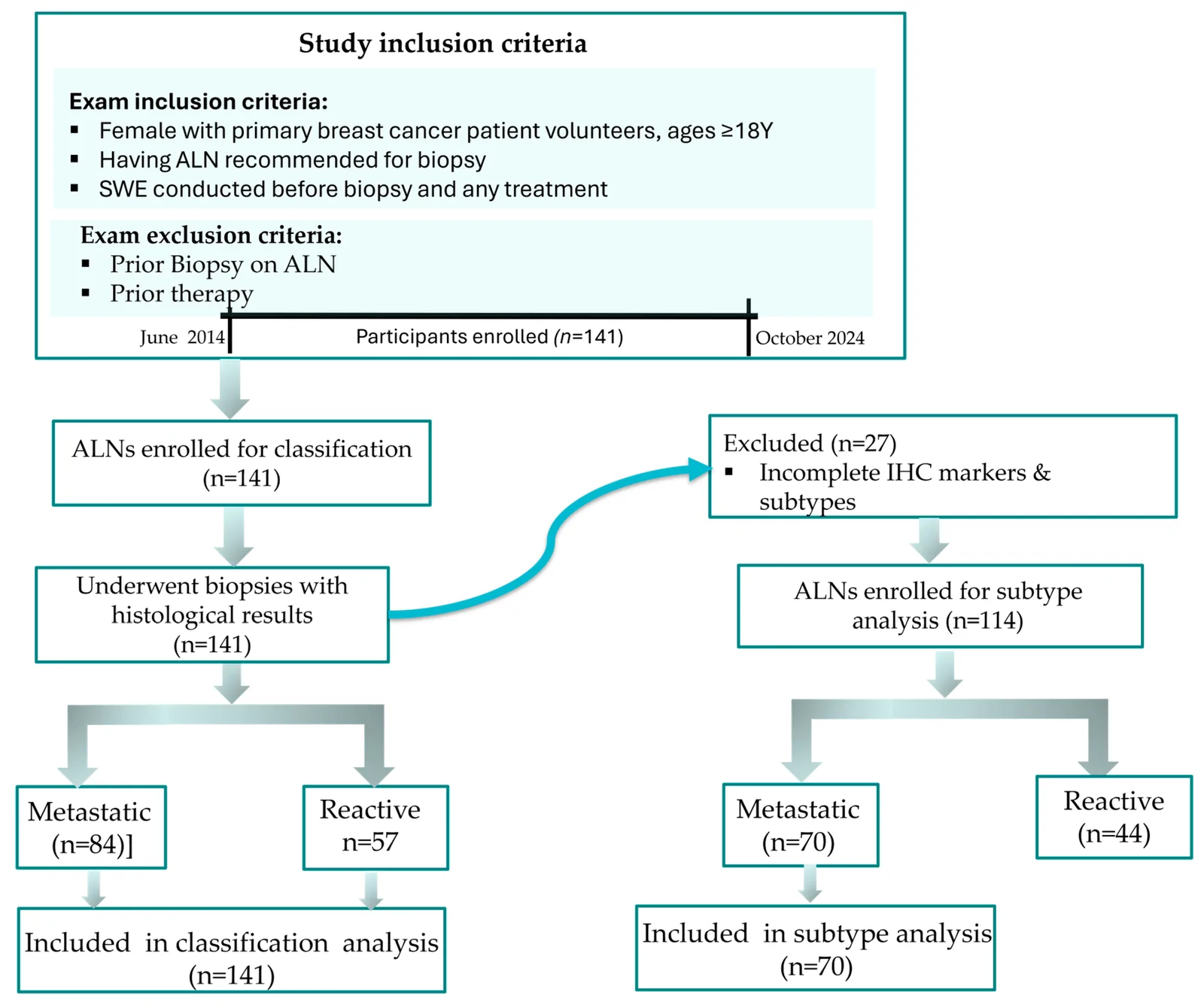
Flow chart of eligibility. Note: ALN = axillary lymph node; SWE = shear wave elastography; IHC = immunohistochemistry.

**Figure 2:**
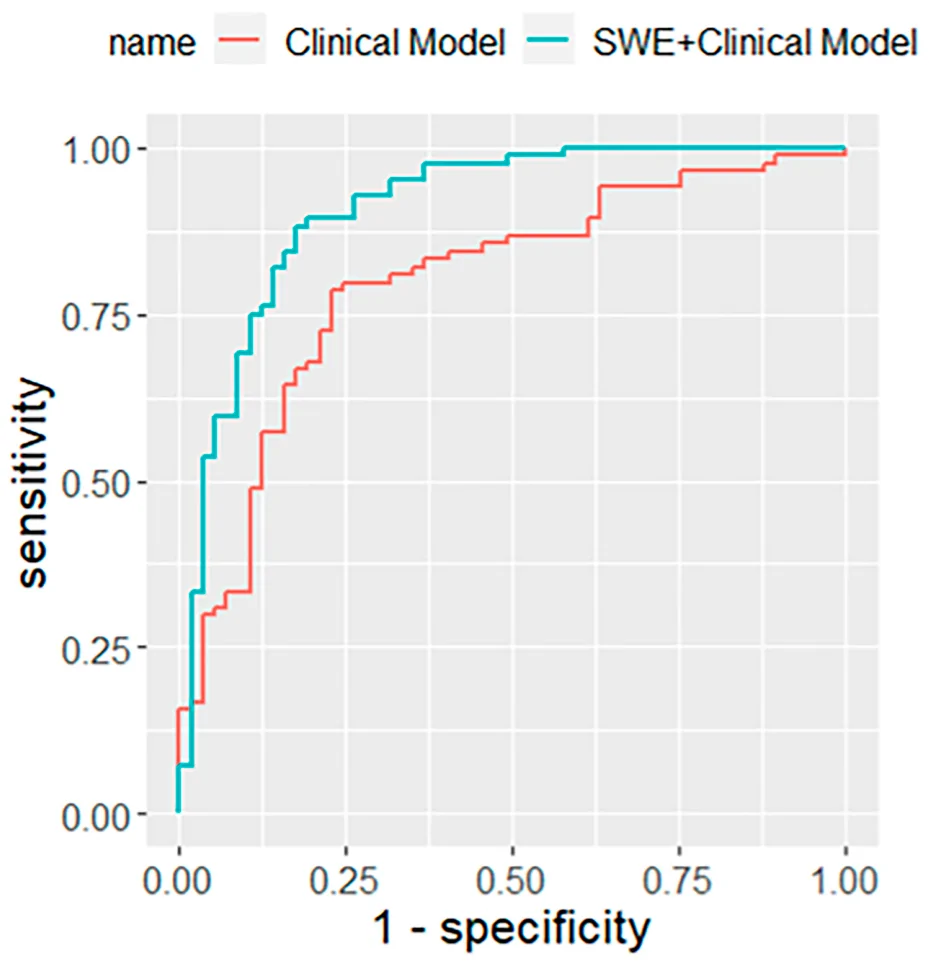
ROC curves for model-based detection of metastatic lymph nodes in breast cancer patients. Note: ROC, receiver operating characteristic; SWE, shear wave elastography.

**Figure 3:**
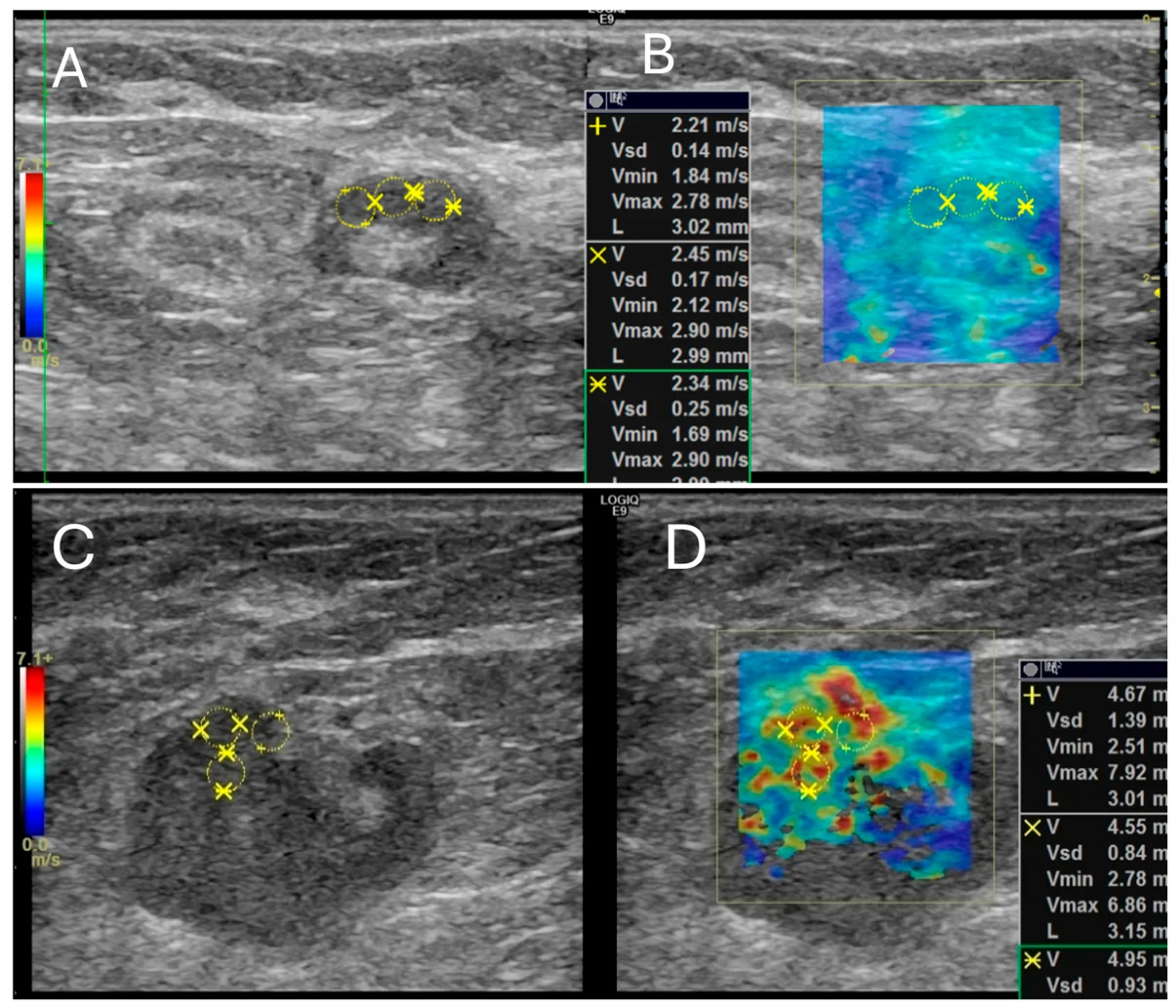
B-mode USs and SWE maps of two larger ALNs—reactive (top) and metastatic (bottom). (**A**,**B**) B-mode US and SWE map of a reactive ALN, with invasive mammary carcinoma as the primary tumor with mixed ductal and lobular features (grade III). The US image displayed an oval, enlarged lymph with a cortical thickness of 7.10 mm and present hilum. The elasticity results calculated from the average of the three ROIs were V_mean_ = 2.33 m/s, V_max_ = 2.86 m/s, V_min_ = 1.88 m/s, V_sd_ = 0.19 m/s, and f_mass_ = 107.62 Hz. Biopsy results indicated a reactive intraparenchymal lymph node. The IHC markers of breast cancer are as follows: ER (−), PR (−), HER2 (−), and Ki-67 = 0.86. The molecular subtype was triple-negative (TN) breast cancer. (**C**,**D**) B-mode US and SWE map of an ALN in a 56-year-old woman with an invasive ductal carcinoma, with focal mucinous features, Nottingham grade II (of III) in her left breast. The ultrasound image showed an oval, enlarged lymph node with a cortical thickness of 21.5 mm and an almost absent hilum. The elasticity results calculated from the average of the three ROIs were V_mean_ = 4.72 m/s, V_max_ = 7.18 m/s, V_min_ = 2.86 m/s, V_sd_ = 1.05 m/s, and f_mass_ = 113.94 Hz. Biopsy results indicated metastatic carcinoma, histopathologically consistent with primary breast cancer. The IHC markers of breast cancer are as follows: ER (+), PR (+), HER2 (−), and Ki-67 = 0.29. The molecular subtype was Luminal B with HER2 Neg.

**Figure 4:**
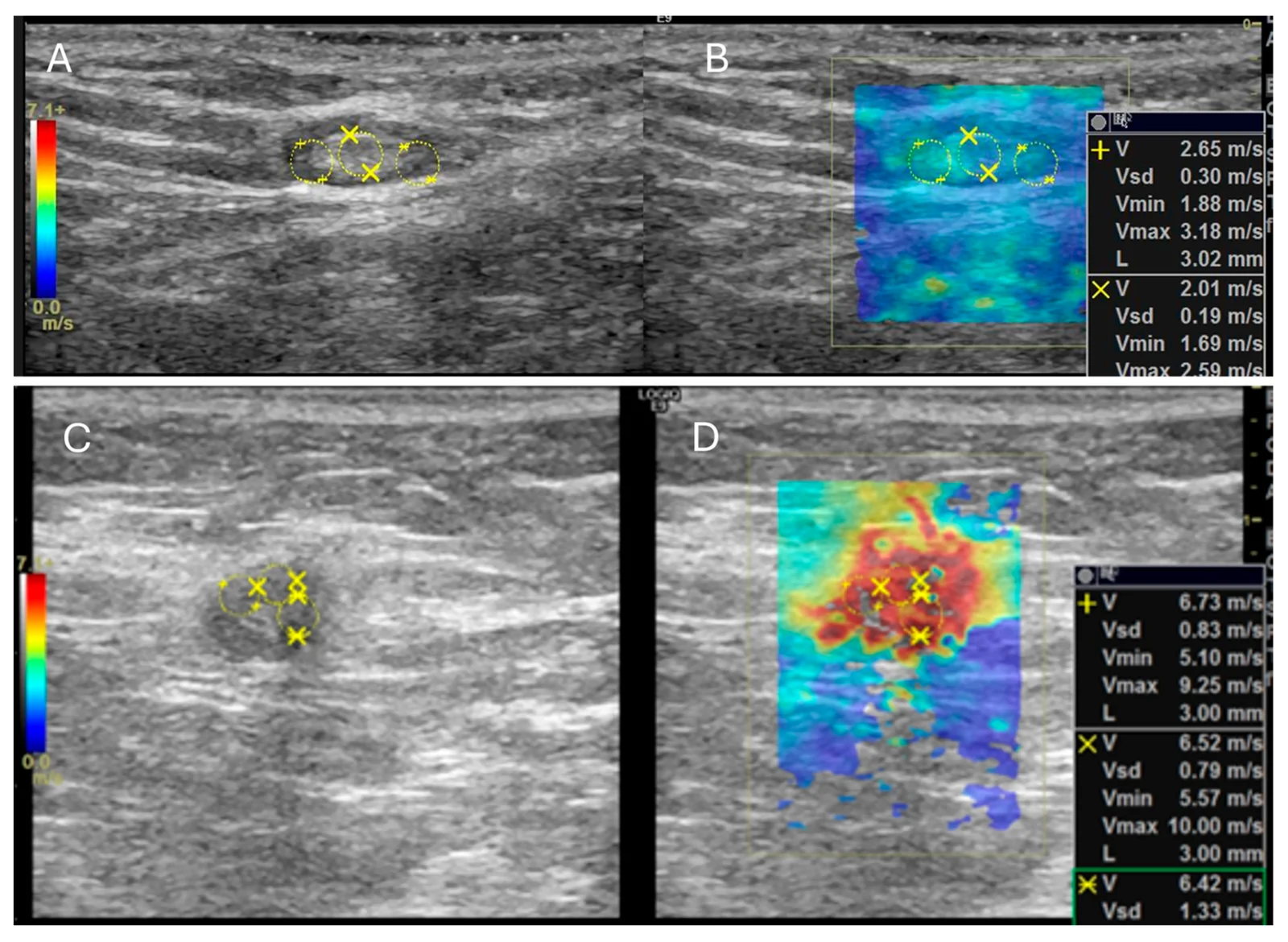
B-mode USs and SWE maps of two smaller ALNs—reactive (top) and metastatic (bottom). (**A**,**B**) B-mode US and SWE map of an ALN with primary breast tumor, Invasive ductal carcinoma, apocrine type, GII, with a cortical thickness of 4 mm and the presence of fatty hilum. The elasticity results calculated from the average of the three ROIs were V_mean_ = 2.16 m/s, V_max_ = 2.76 m/s, V_min_ = 1.52 m/s, V_sd_ = 0.26 m/s and f_mass_ = 82.43 Hz. The IHC markers of breast cancer are as follows: ER (−), PR (−), HER2 (−), and Ki-67 = 0.10. The IHC-defined surrogate subtype was TN. (**C**,**D**) B-mode US and SWE map of an ALN with a primary breast tumor, reported as IDC GII. The ultrasound image shows a lymph node with a cortical thickness of 12.5 mm and an almost absent hilum. The elasticity results calculated from the average of the three ROIs were V_mean_ = 6.56 m/s, V_max_ = 9.46 m/s, V_min_ = 3.80 m/s, V_sd_ = 0.98 m/s, and f_mass_ = 319.89 Hz. Biopsy results indicated metastatic carcinoma, histopathologically consistent with primary breast cancer. The IHC markers of breast cancer are as follows: ER (+), PR (+), HER2 (−), and Ki-67 = 0.315. The IHC-defined surrogate subtype was Luminal B with HER2 Neg.

**Table 1:** Clinical and ultrasound factors of breast cancer patients with ALNs.

Factors	ALNs		Statistical Test	Adj. *p*-Value
	Reactive	Metastatic		
Count	57 (40%)	84 (60%)		
Mean age (y)	58.25 ± 12.00	58.24 ± 12.81	Mann–Whitney U	0.920
Fatty hilum Presence Absence	54 (95%) 3 (5%)	55 (65%) 29 (35%)	Chi-square	0.0009
Cortical thickness (mm) ≤3 >3	4.75 ± 2.14 3 (5%) 54 (95%)	7.27 ± 3.77 3 (4%) 81 (96)	Mann–Whitney U	<0.0001
Shape Oval Round Irregular	54 (95%) 1 (2%) 2 (3%)	53 (63%) 12 (14%) 19 (23%	Chi-square	0.0006
Boundary Clear Unclear	54 (95%) 3 (5%)	54 (64%) 30 (36%)	Chi-square	0.0007

Any *p*-value < 0.0001 was designated as <0.0001.

**Table 2:** Cross-validation classification measures of conventional ultrasonography and SWE in the assessment of reactive and metastatic ALNs with machine learning models.

ML Model	Sens.	Spec	PPV	NPV	Accuracy	AUC (95% CI)
Clinical US	0.786	0.772	0.835	0.710	0.780	0.7980 (0.723–0.873)
SWE + Clinical US	0.881	0.825	0.881	0.825	0.858	0.911 (0.859–0.961)

**Table 3:** Counts and percentages of histological grades and IHC markers, as well as molecular types of breast cancer, in metastatic ALNs (N = 70).

Type	Count
Malignant ALNs	70 (61%)
Malignant grades	
1 2 3	4 (5.7%) 35 (50.0%) 31 (44.3%)
IHC markers	
ER(+)	53 (75.%)
PR(+)	49 (70.0%)
HER2(+)	18 (25.7%)
Ki-67 <14% ≥14%	10 (14.3%) 42 (60.0%)
Missing (did not impact subtype result)	18 (25.7%)
Histological type	
Invasive ductal carcinoma Invasive lobular carcinoma Invasive mammary carcinoma with mixed ducal and lobular features	52 (74.3%) 10 (14.3%) 8 (11.4%)

ER = estrogen receptor; PR = progesterone receptor; HER2 = human epidermal growth factor receptor 2. Numbers in parentheses are percentages of ALN numbers.

**Table 4:** Relationships between SWE and IHC parameters in metastatic lymph nodes (N = 70).

Types (n)	V_mean_ ± SD	V_sd_ ± SD	V_max_ ± SD	V_min_ ± SD	f_mass_ ± SD
IHC subtypes in metastatic nodes (n)					
Luminal A (9)	3.51 ± 1.1	0.74 ± 0.17	5.34 ± 1.25	2.15 ± 1.05	142.6 ± 47.7
Luminal B with HER2+ (11)	4.66 ± 1.17	0.94 ± 0.23	6.92 ± 1.35	2.39 ± 0.96	140.18 ± 79.9
Luminal B with HER2− (35)	3.79 ± 1.45	0.94 ± 0.33	6.15 ± 1.78	1.89 ± 1.18	146.6 ± 109.0
HER2+ (7)	3.31 ± 1.34	0.88 ± 0.34	5.61 ± 1.78	1.57 ± 0.56	104.0 ± 58.8
TN (8)	4.38 ± 1.40	0.93 ± 0.20	6.53 ± 1.50	2.13 ± 0.72	167.4 ± 89.1
IHC-defined surrogate subtype (*p*-value (KW test))	*p* = 0.0250	*p* = 0.0069	*p* = 0.0065	*p* = 0.8783	*p* = 0.9106
Histological grade in metastatic nodes (n)					
1 (4)	4.36 ± 1.49	1.13 ± 0.33	6.95 ± 1.18	1.69 ± 0.85	119.96 ± 69.1
2 (35)	3.80 ± 1.26	0.87 ± 0.27	6.00 ± 1.61	2.04 ± 0.90	151 ± 90.1
3 (31)	3.98 ± 1.54	0.92 ± 0.30	6.23 ± 1.75	1.98 ± 1.22	137.0 ± 97.1
Histological grades (*p*-value (KW test))	*p* = 0.14	*p* = 0.21	*p* = 0.14	*p* = 0.40	*p* = 0.71

ER = estrogen receptor; HER2 = human epidermal growth factor receptor 2; TN = triple negative; IHC = immunohistochemical; SD = standard deviation; SWE = shear wave elastography.

**Table 5:** *p*-Values of pairwise comparisons **of** IHC-defined surrogate subtypes for SWE parameters in metastatic lymph nodes (n = 70).

	Luminal A	Luminal B with HER2+	Luminal B with HER2−	HER2+	TN
Luminal A					
V_mean_	--	0.0018	0.0061	0.33	0.071
V_sd_	--	0.0041	0.0002	0.046	0.20
V_max_	--	0.0009	0.0002	0.095	0.091
Luminal B with HER2+					
V_mean_	0.0018	--	0.63	0.5981	0.86
V_sd_	0.0041	--	1.00	1.00	0.65
V_max_	0.0009	--	1.00	1.00	1.00
Luminal B with HER2-					
V_mean_	0.0061	0.63	--	1.00	1.00
V_sd_	0.0002	1.00	--	1.00	0.46
V_max_	0.0002	1.00	--	1.00	0.90
HER2+					
V_mean_	0.33	0.5981	1.00	--	1.00
V_sd_	0.046	1.00	1.00	--	1.00
V_max_	0.095	1.00	1.00	--	1.00
TN					
V_mean_	0.071	0.86	1.00	1.00	--
V_sd_	0.20	0.65	0.46	1.00	--
V_max_	0.091	1.00	0.90	1.00	--

## Data Availability

The data that support the findings of this study are available from the corresponding author upon reasonable request. The requested data may include figures that have associated raw data. Because the study was conducted on human volunteers, the release of patient data may be restricted by Mayo policy, requiring a special request.

## Abbreviations

The following abbreviations are used in this manuscript:

ALN	Axillary lymph node
CNB	Core needle biopsy
US	Ultrasound
SWE	Shear wave elastography
SD	Standard deviation
Sen	Sensitivity
Spe	Specificity
ROC	Receiver operating characteristic
AUC	Area under the ROC curve
V_mean_	Mean of all measured velocity values
V_max_	Maximum measured velocity value
E_min_	Minimum measured velocity value
V_sd_	Standard deviation of elastography pixel values
PR	Progesterone receptor
HER2	Human epidermal growth factor receptor 2
TN	Triple negative
IHC	Immunohistochemical
NPV	Negative predictive value
PPV	Positive predictive value
2D	Two-dimensional
ROI	Region of interest
LOOCV	Leave-one-out cross-validation
F_mass_	Mass characteristic frequency
FISH	Fluorescence in situ hybridization
